# Mast cell specific receptor Mrgprb2/X2 regulates bladder immunity during urinary tract infections

**DOI:** 10.21203/rs.3.rs-9076947/v1

**Published:** 2026-03-18

**Authors:** Waris Muhammad Khuwaja, Colin Guth, Sudeshna Rakshit, Linghui Nie, Zahra Janjua, Nicole De Nisco, Priyanka Pundir, Dustin Green, Xintong Dong

**Affiliations:** 1 Department of Biological Sciences, University of Texas at Dallas, Richardson, TX 75080, USA; 2 Department of Molecular and Cellular Biology, College of Biological Science, University of Guelph, Guelph, ON N1G 2W1, Canada; 3 Department of Neurobiology, University of Texas Medical Branch, Galveston, TX, USA; 4 Department of Urology, University of Texas Southwestern Medical Center, Dallas, TX, USA

## Abstract

Urinary tract infections (UTIs) are the most common bacterial infections in women. During UTI, the host mounts a rapid immune response to clear the invading pathogen. Mast cells are tissue resident immune cells found in the bladder lamina propria and can serve as first responders to bacterial infections. We investigated the role of the mouse mast cell receptor Mrgprb2 and its human homologue MRGPRX2 in UTI. During acute UTI, Mrgprb2 is activated by the antimicrobial peptide cathelicidin and mediates mast cell degranulation. Using *Mrgprb2* knockout mice, we demonstrated that Mrgprb2 promotes immune cell recruitment and amplifies inflammation, leading to epithelial damage and increased bacterial burden. Pharmacological inhibition of Mrgprb2 with its antagonist osthole improved infection outcomes. Using a humanized *MRGPRX2* knock-in mouse, we show conserved functions of the mouse and human receptors in the bladder. Our findings identify human MRGPRX2 as a potential therapeutic target to improve UTI patient outcomes.

## Introduction

Urinary tract infections (UTIs) are among the most common bacterial infections worldwide, affecting hundreds of millions of individuals each year^1^. Uncomplicated UTIs occur more frequently in women than in men, and additional major risk factors include pregnancy, menopause, and recent sexual activity^1,2^. Although UTIs can be caused by a range of Gram-positive and Gram-negative bacteria, uropathogenic *Escherichia coli* (UPEC) is the predominant etiological agent and is responsible for the highest burden of antimicrobial resistance-associated mortality among UTI-causing pathogens^1,2^. Widespread antibiotic use for UTI treatment has led to the emergence of UPEC strains with increasing resistance to commonly prescribed antibiotics^3^. This growing prevalence of antibiotic-resistant UPEC underscores the urgent need for alternative therapeutic strategies, including immune-modulatory therapies, to enhance host defense against infection.

UTI89 is a well-characterized strain of UPEC isolated from a female cystitis patient that is widely used to model UTI in mice^4^. UTI89 expresses the mannose-binding adhesin FimH, which facilitates bacterial colonization of the bladder by engaging mannose binding pockets on superficial urothelial cells, leading to the formation of intracellular bacterial communities (IBCs)^5,6^. In response to bacterial invasion, the host mounts a robust innate immune response that includes exfoliation of infected superficial urothelial cells to eliminate IBCs. Concurrently, the release of antimicrobial peptides (AMPs) and cytokines from the bladder epithelium work to directly restrict UPEC growth and recruit inflammatory immune cells^7^.The first innate immune cells to respond to UTI in the mouse bladder are neutrophils, followed by a robust type 17 immune response^7,8^. Thus, early epithelial sensing and subsequent immune cell recruitment shape a coordinated immune landscape that determines the outcome of UPEC infection.

Mast cells are tissue-resident immune cells best known for their roles in allergy, but they have also been implicated in host defense against bacterial pathogens^9,10^. Bladder mast cells were previously reported to contribute to exfoliation during acute UTI and pain associated with recurrent UTI and interstitial cystitis^11–13^. The Mas-related G protein-coupled receptor b2 (Mrgprb2), and its human homologue MRGPRX2, are G protein-coupled receptors (GPCRs) expressed by connective tissue mast cells^14^. Mrgprb2+ mast cells are located at host-pathogen interfaces and sense a broad range of ligands, including cationic AMPs, bacterial quorum-sensing peptides, and neuropeptides^15–19^. Mrgprb2/MRGPRX2 activation triggers mast cell degranulation and the release of a large number of inflammatory mediators including histamine, serotonin, lipid mediators and cytokines. These factors increase blood vessel permeability and promote the recruitment of inflammatory immune cells^15,20^. Mrgprb2 has been shown to enhance bacterial clearance in several infection models in the skin, lung, and peritoneal cavity^15–17^. However, the role of Mrgprb2 in bladder immunity remains unknown.

In this study, we use a well-established mouse model of acute cystitis to examine the role of Mrgprb2 and MRGPRX2 in UTI. We found that Mrgprb2/MRGPRX2 negatively impact UPEC cystitis outcomes by promoting a maladaptive inflammatory response and exacerbating urothelial damage. Pharmacological inhibition of Mrgprb2 with osthole, a preclinical antagonist, significantly reduced bladder bacterial burden. Our findings identify MRGPRX2 as a potential therapeutic target for limiting bladder pathology and improving patient outcomes during UTI.

## Results

### Mrgprb2+ mast cells are found in the bladder and impair UPEC clearance during UTI

**Figure 1:**
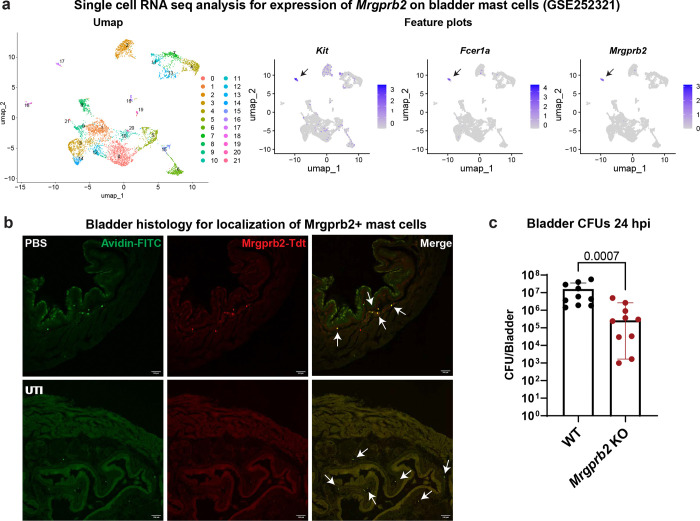
Mrgrb2+ mast cells in the bladder exacerbate UTI **a**) Umap and feature plots from previously published single cell RNA sequencing (GSE252321) showing that Mrgprb2+ mast cells form a distinct cluster that expresses *Kit*, *Fcer1a*, and *Mrgprb2*. **b**) Representative histology images showing the location of Mrgprb2+ mast cell (arrows) within bladder under PBS and UTI condition. Green is Avidin stain, and red is Mrgprb2^*TdTomato*^. Scale bar is 100μm. **c**) Bacterial CFUs in bladders of WT and *Mrgprb2* KO mice 24 hpi. Graph shows median with 95% confidence interval (CI). WT and *Mrgprb2* KO n = 10. p value is calculated by two-tailed Mann-Whitney U test.

Previous studies have defined two major mast cell subsets: Mrgprb2-positive (Mrgprb2+) mast cells, which are enriched in connective tissues, and Mrgprb2-negative (Mrgprb2-) mast cells, which are found in the digestive tract^14^. To determine whether bladder mast cells express Mrgprb2, we analyzed a previously published single-cell RNA sequencing dataset of mouse bladder immune cells^21^. This analysis revealed a distinct cluster (cluster # 17) expressing *Mrgprb2*, as well as the c-Kit receptor for stem cell factor (*Kit*), and the high affinity IgE receptor FcεR1 (*Fcer1a),* consistent with a Mrgprb2+ mast cell identity^22^ (Fig. 1a).

To independently validate the presence and anatomical localization of Mrgprb2+ mast cells in the bladder, we used an *Mrgprb2*^*Tdt*^ reporter mice^23^. Bladders were collected from mice 24 hours post transurethral inoculation with vehicle (PBS) or with UPEC strain UTI89, sectioned, and stained with Avidin-FITC to label mast cells^24^. Histological analysis confirmed the presence of Mrgprb2+ mast cells within the bladder lamina propria in naïve and infected mice (Fig. 1b).

Having established the presence of Mrgprb2+ mast cells in the bladder, we next examined their role during acute UTI. Based on prior reports implicating Mrgprb2+ mast cells in antibacterial host defense^16^, we initially hypothesized that these cells would promote clearance of uropathogenic *Escherichia coli* (UPEC). To test this, 7–10-week-old female wild-type C57BL/6J (WT) and *Mrgprb2* knockout (KO) mice^23^ were infected with UPEC strain UTI89, and bacterial burden was assessed in bladders 24 hours post-infection (hpi). Converse to our initial hypothesis, *Mrgprb2* KO mice exhibited ~2-log reduction in bladder UPEC CFUs compared with WT controls (Fig. 1c). No differences in kidney CFUs were observed between the two genotypes at 24 hpi (Fig. S1). Together, these data demonstrate that Mrgprb2+ mast cells reside within the bladder and, during acute UTI, limit efficient clearance of UPEC from the bladder.

### Mrgprb2 regulates transcription of host immunity-related genes during UTI

**Figure 2:**
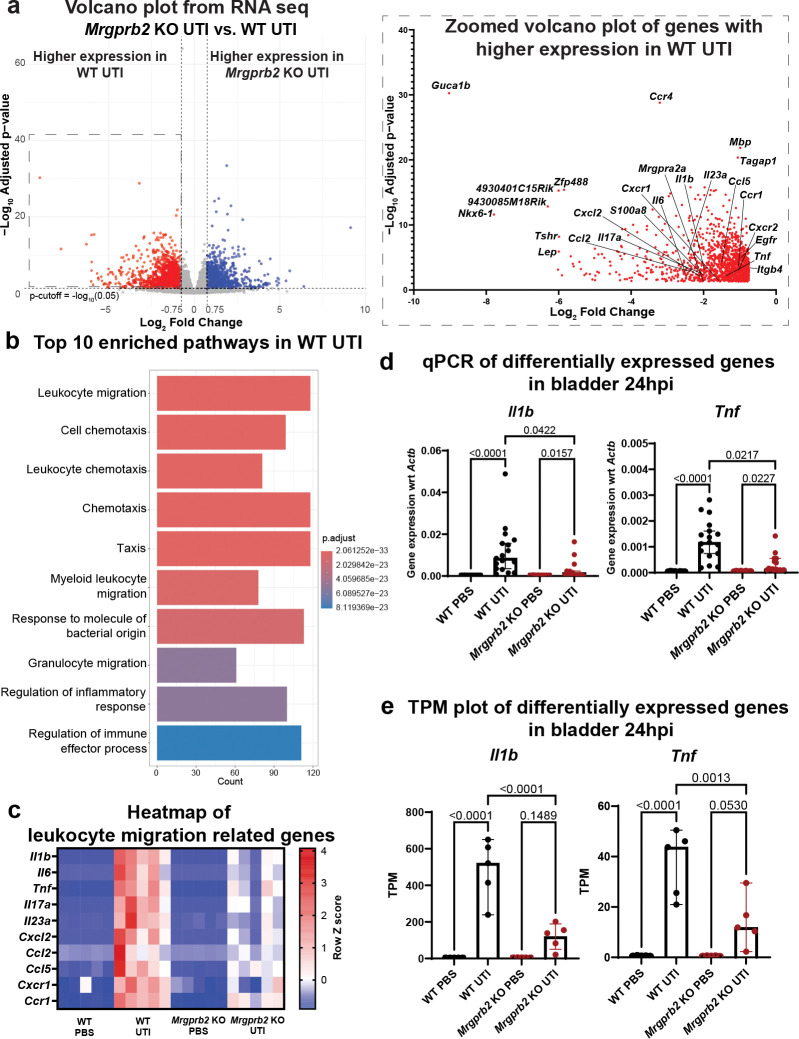
Mrgprb2 promotes transcriptional changes related to leukocyte migration and inflammation in bladder post UTI **a**) Volcano plot showing differentially expressed genes in WT UTI versus *Mrgprb2* KO UTI bladders 24 hpi. Log_2_foldchange > 0.75 represent genes with higher expression in *Mrgprb2* KO UTI bladder and Log_2_foldchange < −0.75 represents genes with higher expression in WT UTI bladders. p-value cut off is -log_10_(0.05). Zoomed in view of the volcano plot shows genes higher expressed in WT UTI group with relevant genes for this study labelled. **b**) Bar plot showing top 10 enriched pathways in the gene set that was significantly higher in WT UTI compared to *Mrgprb2* KO UTI. Gene ontology (GO) analysis was performed using ClusterProfiler. **c**) Heatmap showing the expression of genes in the GO pathway leukocyte migration in WT PBS, WT UTI, *Mrgprb2* KO PBS, *Mrgprb2* KO UTI samples. **d**) qPCR results showing expression of *Il1b* and *Tnf* with respect to *Actb* in PBS-treated and post-UTI WT and *Mrgprb2* KO mice. Graph shows median with 95% CI. Kruskal-Wallis test is performed to calculate p-values. **e**) Transcripts Per Million (TPM) values from RNA sequencing data of *Il1b* and *Tnf* in PBS and UTI conditions in WT and *Mrgprb2* KO mice. Graph shows median with 95% CI. p-values are calculated using ordinary one-way ANOVA.

To understand how Mrgprb2 regulates bacteria clearance in the bladder, we performed bulk RNA sequencing (RNAseq) on WT and *Mrgprb2* KO bladders 24 hpi with UPEC UTI89 or with PBS vehicle control. RNAseq analysis of host transcripts revealed transcriptional differences between infected WT and *Mrgprb2* KO bladders, whereas minimal differences were observed between PBS-treated groups (Fig. 2a and Fig. S2a-b). Differential gene expression analysis on this dataset identified a distinct set of genes that were expressed at higher levels in WT bladders 24 hours post UPEC infection compared to *Mrgprb2* KO bladders (Fig. 2a). Gene Ontology (GO) enrichment analysis of these genes, performed using ClusterProfiler^25^, revealed significant enrichment of biological processes related to leukocyte migration, cell chemotaxis, response to molecule of bacterial origin, and regulation of inflammatory response (Fig. 2b–c, Fig. S2c). These pathways included pro-inflammatory cytokines such as *Il1b* and *Tnf* (Fig. 2c).

To validate the RNAseq findings, we performed quantitative-PCR (qPCR) on RNA isolated from bladders of WT and *Mrgprb2* KO mice infused with PBS or infected with UTI89 for 24 hours. Consistent with the RNAseq results, expression of the proinflammatory cytokines *Il1b* and *Tnf* was markedly increased in infected WT bladders relative to PBS controls (424-fold for *Il1b* and 33-fold for *Tnf*). In contrast, compared to PBS controls, infected *Mrgprb2* KO bladders showed only a 33-fold increase in *Il1b* expression (a 12-fold decrease compared to WT) and a 3.5-fold increase in *Tnf* expression (an 8-fold decrease compared to WT) (Fig. 2d–e). Together, these data indicate that Mrgprb2 alters bladder immunity-related gene transcription during UPEC UTI, particularly pathways associated with leukocyte migration and inflammation.

### Mrgprb2+ mast cells promote immune cell infiltration into the bladder during UTI

**Figure 3:**
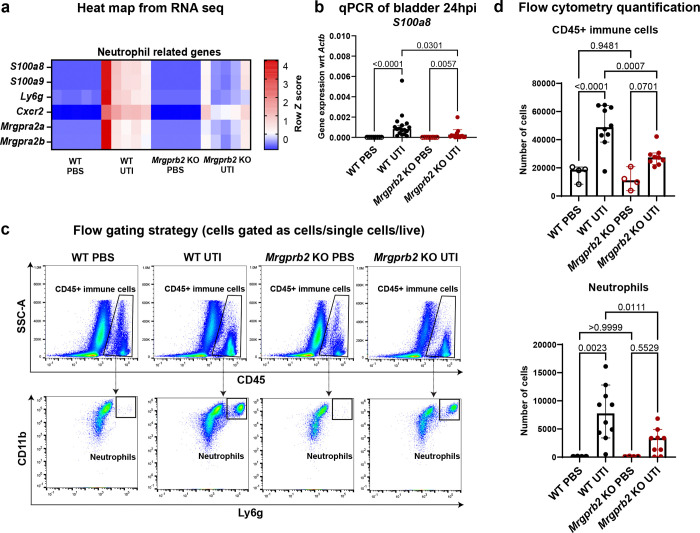
Mrgpb2 promotes immune cell, particularly neutrophil, infiltration into bladder during UTI **a**) Heatmap from RNAseq data showing expression of neutrophil related genes in WT and *Mrgprb2* KO bladders in PBS and UTI conditions. **b**) qPCR analysis showing expression of *S100a8* gene with respect to *Actb* in WT and *Mrgprb2* KO bladders in PBS and UTI conditions 24 hpi. Graph shows median with 95% CI. p-values are calculated using Kruskal-Wallis test. **c**) Flow cytometry plots showing representative gates for CD45+ immune cells and neutrophils. **d**) Flow cytometry quantification showing the changes in number of CD45+ immune cells and neutrophils in WT and *Mrgprb2* KO bladders in PBS and UTI condition 24 hpi. Graph shows median with 95% CI. p-values are calculated using ordinary one-way ANOVA.

Bulk RNAseq analysis of mouse bladders indicated that Mrgprb2 is associated with transcriptional changes linked to leukocyte migration during UPEC UTI. Neutrophils are among the earliest immune responders recruited to the bladder during infection^7^. Based on these transcriptomic findings and the established role of Mrgprb2 in promoting immune cell recruitment at sites of injury and infection^16,26^, we hypothesized that immune cell infiltration, particularly neutrophil recruitment post infection will be reduced in *Mrgprb2* KO bladders compared to WT bladders. To test this, we first examined the expression of neutrophil-associated marker genes in the bulk RNAseq dataset. Differential expression analysis of canonical neutrophil marker genes (*S100a8*, *S100a9*, *Ly6g*, *Cxcr2*, *Mrgpra2a*, and *Mrgpra2b*)^27–29^ showed a strong increase in their expression in WT bladders following infection, whereas only a slight increase was observed in infected *Mrgprb2* KO bladders. Compared with WT infected bladders, *Mrgprb2* KO infected bladders exhibited ~50% lower expression of these transcripts (Fig. 3a, Fig. S3a).

These findings were further validated by qPCR analysis. *S100a8* expression increased markedly (~82-fold) in WT bladders following infection. Infection also induced *S100a8* expression in *Mrgprb2* KO bladders but only by ~14-fold relative to PBS-treated controls. The magnitude of infection-induced *S100a8* expression was significantly lower in *Mrgprb2* KO mice, with an overall ~5-fold reduction compared with WT bladders. (Fig. 3b, Fig. S3a).

To directly assess immune cell infiltration into the bladder during UTI, we performed flow cytometry analysis on bladders from WT and *Mrgprb2* KO mice transurethrally inoculated with PBS and UTI89 24hpi. Immune cells were identified as Live/CD45+ cells, and neutrophils were identified as Live/CD45+/CD11b+/Ly6g+ cells (Fig. 3c, Fig. S3b). Consistent with the bulk RNAseq and qPCR results, UPEC infection increased total immune cell infiltration in WT bladders from a median of 18,608.5 cells in the PBS group to a median of 48,740.5 cells in the UTI group. Neutrophil numbers increased from a median of 43.5 cells in the PBS group to 7,735.5 cells in the UTI group. In contrast, infected *Mrgprb2* KO bladders exhibited significantly fewer immune cells (median of 11,056.5 cells) and neutrophils (median of 3,362 cells) compared with infected WT bladders (Fig. 3d). Collectively, these data demonstrate that Mrgprb2 promotes immune cell, particularly neutrophil, infiltration into the bladder during UPEC UTI.

### Mrgprb2-mediated bladder inflammation drives epithelial damage during UTI

Our findings thus far established a role for Mrgprb2 in promoting immune cell infiltration into the bladder and inflammation during UPEC UTI. Although inflammation is generally considered beneficial for bacterial control, excessive inflammation can lead to bladder epithelial damage and exacerbate infection^21,30,31^. Based on these past observations, we hypothesized that *Mrgprb2* KO mice experience reduced bladder epithelial damage during acute UTI.

**Figure 4:**
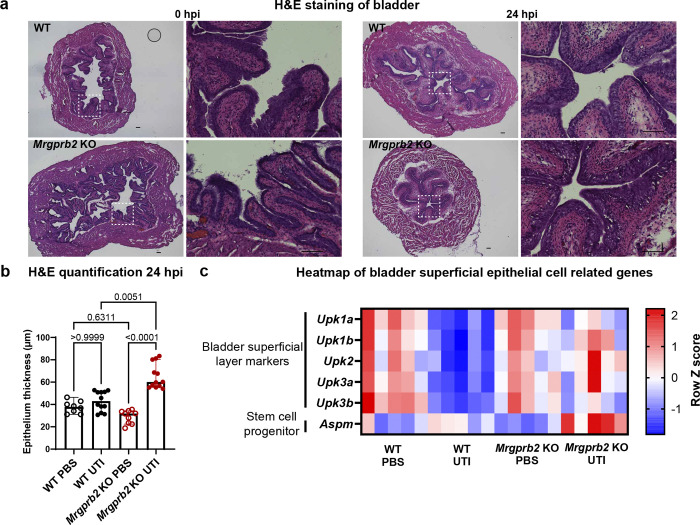
Mrgprb2 regulates urothelial regeneration during UTI **a)** Representative images showing WT and *Mrgprb2* KO bladders stained with H&E in PBS and UTI conditions 24 hpi. White dotted box shows the area zoomed in. Scale bars are 100μm. **b)** Quantification of the H&E images from **a** showing epithelial thickness (inμm) in PBS and UTI conditions in WT and *Mrgprb2* KO mice. Graphs show median with 95% CI. p-values are calculated using Kruskal-Wallis test. **c)** Heatmap from RNAseq data showing expression of uroplakin genes and stem cell progenitor *Aspm* in WT and *Mrgpb2* KO mice in PBS and UTI conditions.

To test this hypothesis, WT and *Mrgprb2* KO mice were transurethrally inoculated with PBS or UPEC UTI89 for 24 hours. Bladder tissues were then collected and analyzed by hematoxylin and eosin (H&E) staining. No obvious differences in epithelial morphology were observed between genotypes under PBS-treated conditions. However, at 24 hpi, the *Mrgprb2* KO bladder epithelium appeared markedly thicker than both the PBS control and infected WT bladders (Fig. 4a). Quantification of epithelial thickness confirmed that the bladder epithelium of UPEC-infected *Mrgprb2* KO mice (median, 60.0 μm) was ~32 μm thicker than PBS-treated *Mrgprb2* KO controls (median, 31.7 μm) and ~15 μm thicker than infected WT mice (median, 42.9 μm) (Fig. 4b). No significant differences in epithelial thickness were observed between infected and uninfected WT groups. These findings suggest that *Mrgprb2* KO mouse has thicker epithelium post infection due to attenuated inflammation.

To determine whether these histological changes were reflected at the transcriptional level, we interrogated our bulk RNAseq data for genes associated with bladder epithelial markers, and epithelial regeneration. The expression of genes encoding uroplakins that constitute the superficial and intermediate urothelial layers (*Upk1a*, *Upk1b*, *Upk2*, *Upk3a*, *Upk3b*)^32^ were reduced in WT and *Mrgprb2* KO bladders following infection. However, the reduction was approximately 2-fold greater in WT mice than in *Mrgprb2* KO mice, consistent with increased loss of superficial urothelial cells in WT bladders after infection (Fig. 4c, Fig. S4). In addition, the expression of stem cell progenitor-associated gene *Aspm*^33^ remained unchanged in WT bladders 24 hours after infection, whereas it was about 3-fold higher in *Mrgprb2* KO bladders, consistent with thicker epithelium and better repair. There was an overall ~1.7-fold reduction in *Aspm* transcripts in WT infected bladders when compared to *Mrgprb2* KO infected bladders (Fig. 4c, Fig. S4). Collectively, these results indicate that Mrgprb2-mediated inflammation during UTI may contribute to bladder epithelial damage.

### Mrgprb2 enhances bladder epithelial receptivity to UPEC invasion during UTI

**Figure 5:**
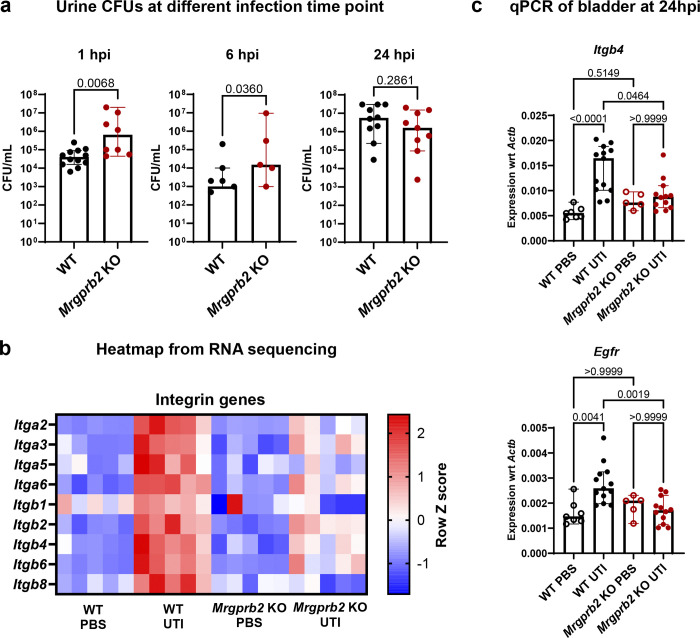
Mrgprb2 regulates epithelial receptivity that promotes UPEC invasion during UTI **a**) UTI89 CFUs in urine of WT and *Mrgprb2* KO mice 1, 6 and 24 hpi. Graph shows median with 95% CI. p-values are calculated using two-tailed Mann-Whiteny U test. **b**) Heatmap from RNAseq data showing expression of integrin subunits in bladders of WT and *Mrgprb2* KO mice in PBS and UTI condition. **c**) qPCR analysis showing expression of *Itgb4* and *Egfr*, normalized to *Actb*, in WT and *Mrgprb2* KO mice bladder pre and 24 hours post infection. Graph shows median with 95% CI. p-values are calculated using Kruskal-Wallis test.

To monitor the progression of bladder infection in WT and *Mrgprb2* KO mice, we collected urine at 1, 6, and 24 hpi and quantified bacterial burden by CFU enumeration. *Mrgprb2* KO mice exhibited ~2-log higher urine UPEC burden at 1 hpi and ~1-log higher urine UPEC burden at 6 hpi compared with WT mice (Fig. 5a). We hypothesized that the elevated urine UPEC burden at these early time points may reflect reduced bacterial attachment in *Mrgprb2* KO bladders during the initial stages of infection, which could subsequently contribute to lower bacterial burdens at 24 hpi. Based on these observations, we next interrogated the RNAseq dataset to identify host genes that may facilitate UPEC attachment.

Integrins are critical regulators of bladder epithelial integrity and epithelial receptivity during UTI. Several integrin subunits, including ITGB4, ITGB1, ITGA2, ITGA3, and ITGA6, have been identified as host receptors for the UPEC adhesin FimH, facilitating bacterial binding and attachment^34^. To determine whether Mrgprb2 influences integrin expression during UTI, we examined the expression of integrin genes in our bulk RNAseq dataset. Heatmap analysis revealed that gene expression of multiple integrin subunits (*Itgb1*, *Itgb2*, *Itgb4*, *Itgb6*, *Itgb8*, *Itga2*, *Itga3*, *Itga5*, and *Itga6*) increased in WT mice by about 2-fold upon infection whereas expression remained unchanged in *Mrgprb2* KO mice (Fig. 5b, Fig. S5). We validated these findings by qPCR, which confirmed the expression of *Itgb4* in infected WT bladders increased by 3-fold compared to PBS controls, but no changes were observed in *Mrgprb2* KO bladders (Fig. 5c).

Previous studies have also implicated epidermal growth factor receptor (EGFR) signaling in promoting UPEC invasion *in vitro*^35^. To determine whether EGFR expression was altered during UPEC bladder infection *in vivo*, we measured *Egfr* transcript levels in bladders with qPCR and our RNAseq data. qPCR analysis showed that *Egfr* expression increased approximately ~1.8-fold in infected WT bladders but remained unchanged in infected *Mrgprb2* KO bladders (Fig. 5c). Consistent with these findings, RNAseq analysis also revealed a similar ~2-fold increase in *Egfr* transcripts in WT bladders following infection, whereas no change was observed in *Mrgprb2* KO bladders (Fig. S5). Collectively, these data suggest that Mrgprb2 could promote bladder epithelial receptivity to UTI89 during UTI by upregulating integrins and EGFR-associated pathways, thereby facilitating UPEC attachment at early stages of infection.

### Mrgprb2 is activated by the antimicrobial peptide cathelicidin during UTI

Mrgprb2 recognizes diverse cationic ligands, including neuropeptides, compound 48/80, and AMPs such as cathelicidin^15,18,19^ (whose mature form is called LL-37 in humans; and Cathelicidin-Related Antimicrobial Peptide or CRAMP in mice^36^). Interestingly, prior work by Danka *et al.*^37^ showed that *Camp* KO mice lacking cathelicidin displayed similar phenotypes as *Mrgprb2* KO mice in terms of UPEC CFUs, inflammation, and epithelial damage during UTI89 induced UTI, suggesting that cathelicidin and Mrgprb2 may function within the same pathway to shape bladder immunity. Given this past work, we sought to test whether cathelicidin signals through Mrgprb2 to drive bladder pathology during UTI.

To directly test whether cathelicidin signals through Mrgprb2 to induce mast cell degranulation, we stimulated peritoneal cavity-derived mast cells (PCMCs) from WT and *Mrgprb2* KO mice with vehicle, CRAMP (1 μM or 10 μM), or compound 48/80 (positive control). Degranulation was quantified by β-hexosaminidase release. WT PCMCs exhibited robust degranulation in response to 10 μM CRAMP and compound 48/80, whereas vehicle control induced minimal release. In contrast, *Mrgprb2* KO PCMCs failed to degranulate in response to either CRAMP or compound 48/80 (Fig. S6a).

To determine whether this interaction is conserved in humans, we stimulated WT and *MRGPRX2* KO LAD2 mast cells with LL-37. WT cells exhibited dose-dependent degranulation at 5 and 10 μM LL-37, whereas *MRGPRX2* KO cells showed no changes in β-hexosaminidase release at any concentration tested (Fig. S6b). These findings demonstrate that cathelicidin-induced mast cell degranulation requires Mrgprb2/X2, supporting a direct functional interaction between cathelicidin and Mrgprb2/MRGPRX2.

Given the previous report of cathelicidin in UTI89 induced UTI, we next assessed whether *Camp* KO mice recapitulate the bacterial burden observed in *Mrgprb2* KO mice during UTI. To test this, 7 to 10-week-old female WT, *Mrgprb2* KO, and *Camp* KO mice were infected with UTI89, and bladder CFUs were quantified 24 hpi. As expected, both *Mrgprb2* KO and *Camp* KO mice exhibited an approximately 2-log reduction in bladder CFUs compared to WT controls. Notably, CFU levels did not differ between *Mrgprb2* KO and *Camp* KO mice (Fig. S6c). There was no difference observed in kidney CFUs between the three groups (Fig. S6d).

Together, the UTI89 CFU phenotype similarity between *Camp* KO and *Mrgprb2* KO mice, along with the Mrgprb2/X2 dependent mast cell degranulation in response to cathelicidin observed *in vitro*, supports a model in which cathelicidin acts upstream of Mrgprb2 to modulate responses that exacerbate UPEC UTI.

### Targeting human MRGPRX2 as a potential therapeutic strategy to treat UTI

**Figure 6:**
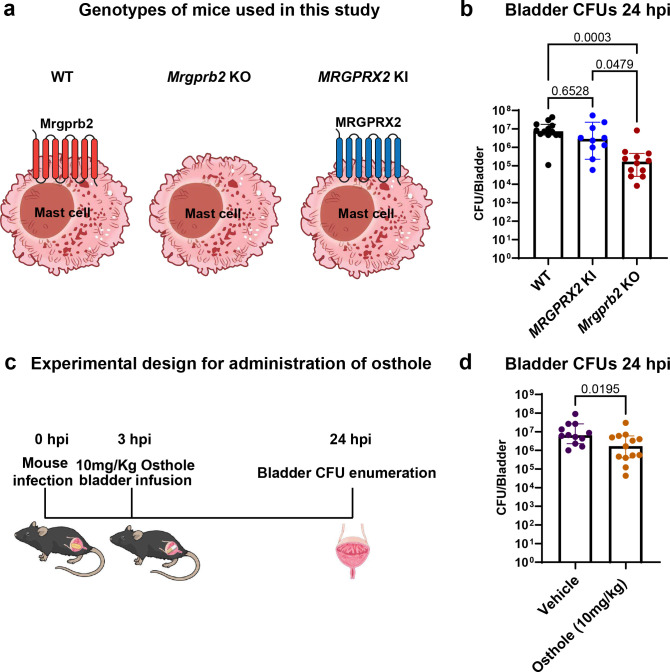
MRGPRX2 serves as therapeutic target to improve UTI **a)** Schematics showing mast cells in WT, *Mrgprb2* KO, and *MRGPRX2* KI mice used in this study. **b)** Bladder CFUs 24 hpi in WT, *MRGPRX2* KI, and *Mrgprb2* KO mice. Graph shows median with 95% CI. p-values are calculated using Kruskal-Wallis test. **c)** Schematic showing experimental outline for osthole delivery into the mice. **d)** Bladder CFUs 24 hpi in WT mice treated with vehicle or 10mg/kg osthole. Graph shows median with 95% CI. p-values are calculated using two-tailed Mann-Whitney U test.

Having established the role of mouse Mrgprb2 in regulating bladder immunity during UTI, we next sought to assess the translational relevance of our findings by first assessing the functional homology between mouse Mrgprb2 and its human homologue MRGPRX2. To do this, we utilized a humanized *MRGPRX2* knock-in (KI) mouse in which *Mrgprb2* is knocked out and instead, *MRGPRX2* is expressed on mast cells under the control of *Mrgprb2*-cre (*Mrgprb2-cre; Rosa26-lsl-MRGPRX2; Mrgprb2* KO mice) ^23^ (Fig. 6a). Given the well-established homology between Mrgprb2 and MRGPRX2^15^, we hypothesized that the UPEC bladder CFU phenotype observed in *Mrgprb2* KO mice would be rescued in the *MRGPRX2* KI mice.

To test this, WT, *Mrgprb2* KO, and *MRGPRX2* KI mice were infected with UTI89 for 24 hours, and bladder bacterial burdens were assessed. As expected, *Mrgprb2* KO mice exhibited ~2-log lower bladder CFUs compared with WT and *MRGPRX2* KI mice, whereas *MRGPRX2* KI mice displayed CFUs comparable to WT, indicating rescue of the phenotype (Fig. 6b). No differences were observed in kidney bacterial burdens among the three groups (Fig. S7a).

We next examined whether pharmacological inhibition of Mrgprb2 could reduce bladder bacterial burden in WT mice. To test this, we infected WT mice with UTI89 and at 3 hpi, infused bladders with 10 mg/Kg of the Mrgprb2 antagonist osthole^38^ or vehicle control via transurethral catheterization (Fig. 6c). Osthole-treated mice exhibited ~1-log reduction in bladder UPEC burden compared with vehicle-treated controls (Fig. 6d). Minimum inhibitory concentration (MIC) assay confirmed that osthole at the administered dose had no direct antibacterial effect on UTI89 (Fig. S7b), indicating that the reduction in bacterial burden was mediated via Mrgprb2 inhibition rather than direct bacterial killing.

Collectively, these results demonstrate that the role of Mrgprb2 and MRGPRX2 in promoting bladder infection is conserved and that pharmacological inhibition of Mrgprb2 can reduce UPEC burden. These findings suggest that targeting MRGPRX2 may serve as a novel therapeutic strategy to improve patient outcomes during UTI.

## Discussion

Our study identifies a previously unrecognized role for mast cells in exacerbating UPEC infection and modulating epithelial repair following UTI. While prior studies have largely focused on Mrgprb2’s function in skin infections, where it promotes protective immune responses and enhances pathogen clearance^16^, we demonstrate, for the first time, its role in a bladder infection. Notably, although mast cell-driven inflammation is similarly amplified in the bladder, the consequence for bacterial burden diverges from observations in the skin, highlighting tissue-specific outcomes of Mrgprb2 signaling during host-pathogen interactions.

**Figure 7:**
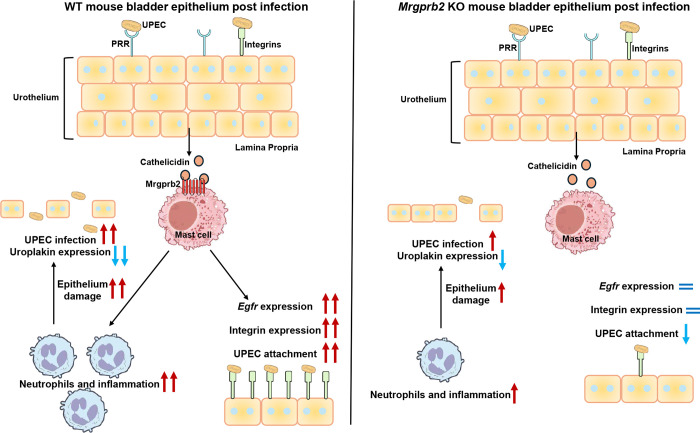
Summary of major findings Schematic model illustrating the proposed mechanism of cathelicidin-Mrgprb2 signaling during UTI. In WT mice **(left)**, cathelicidin binding to Mrgprb2 enhances inflammatory responses and increase integrin and *Egfr* expression, thereby promoting epithelial damage, bacterial attachment, and infection. In contrast, in *Mrgprb2* KO mice **(right)**, absence of Mrgprb2 attenuates inflammation, thereby limiting bacterial infection and infection-associated epithelial damage.

Based on our findings, we propose the following model (Fig. 7): In WT mice, bladder epithelial cells (BECs) sense UPEC through pattern recognition receptors (PRRs), leading to the release of cathelicidin. Cathelicidin, in turn, activates Mrgprb2 on mast cells, triggering degranulation. Mediators released from mast cells promote recruitment of innate immune cells, including neutrophils, and may drive upregulation of integrins in bladder epithelial cells, thereby enhancing epithelial adhesion to UPEC and amplifying local inflammation. While inflammatory responses are typically protective, our data indicate that excessive Mrgprb2-dependent inflammation in WT mice results in reduced epithelial thickness, thereby facilitating UPEC infection. In contrast, in *Camp* KO or *Mrgprb2* KO mice, cathelicidin-Mrgprb2 signaling is abolished, resulting in no mast cell degranulation. Consequently, inflammation is attenuated, and epithelium thickness is increased.

We also observed reduced integrin expression in *Mrgprb2* KO mice, which may correlate with diminished UPEC attachment. This is reflected by higher urine CFUs at early time points, consistent with decreased bacterial attachment and enhanced clearance in urine. In parallel, whereas *Egfr* expression is highly upregulated in WT mice, matching prior reports linking EGFR signaling to UPEC invasion^35^, *Mrgprb2* KO mice showed no infection-induced changes in *Egfr* expression. These data indicate that Mrgprb2 signaling promotes a transcriptional program that enhances epithelial receptivity to UPEC. We therefore propose that Mrgprb2 regulates epithelial changes during UTI, such that, in WT mice, it promotes an inflammatory and adhesive signal that supports immune cell recruitment but allows bacterial attachment, whereas in *Mrgprb2* KO mice, reduced integrin-mediated adhesion triggers proliferative signaling while limiting pathogen entry.

Our findings also provide a mechanistic explanation for prior observations in *Camp* KO mice. Danka *et al.* reported the surprising finding that during UTI89 induced UTI, *Camp* KO mice which lacks a key antimicrobial peptide, displayed reduced UPEC CFUs, attenuated inflammation, and less epithelial damage, phenotypes we similarly observe in *Mrgprb2* KO mice^37^. Our study indicates that these effects arise from loss of ligand-driven Mrgprb2 activation. These observations may extend beyond UTI89, as studies with Group B *Streptococcus* too shows that cathelicidin can exacerbate bladder infection^39^. Future studies are needed to understand the significance of Mrgprb2/cathelicidin signaling in other bladder-pathogen interactions.

Importantly, we demonstrate that the phenotypes observed with mouse Mrgprb2 are conserved in the human homologue, MRGPRX2. Pharmacologic inhibition of Mrgprb2 signaling using the preclinical compound osthole reduced bacterial burden following infection, supporting the therapeutic potential of targeting the Mrgprb2/MRGPRX2 axis. In the context of rising antibiotic resistance, host-directed therapies that inhibit MRGPRX2 signaling may offer a promising alternative for UTI treatment. Together, these findings establish MRGPRX2 as a potential drug target for UTI.

Future studies are needed for several findings reported here. First, although we observe rapid epithelial proliferation in infected *Mrgprb2* KO mice, the cellular origin of these proliferating cells remains undefined. While differentiation markers, stem cell-associated genes, and uroplakin transcripts are upregulated, lineage tracing of these cells is beyond the scope of the current study. Second, we plan to perform single cell RNAseq on immune cells from WT and *Mrgprb2* KO mice to comprehensively profile immune cell dynamics during UTI, extending beyond neutrophils. Third, our analysis primarily focuses on the 24-hour post-infection time point. A more detailed study, including earlier stages such as 1, 3, and 6 hours post-infection, will provide deeper insight into the kinetics of inflammatory and epithelial responses. Finally, while this study focuses on the host response, future work will investigate how UPEC transcriptional programs adapt to alterations in the host environment in the absence of Mrgprb2.

Overall, our findings redefine mast cell function in UTI, revealing that Mrgprb2 activation drives inflammation while simultaneously making the epithelium more permissive to UPEC infection. Targeting the Mrgprb2/MRGPRX2 axis may thus represent a promising strategy to limit bacterial burden and enhance tissue repair during UTI.

## Methods

### Mice

All animal experiments were approved by the Institutional Animal Care and Use Committee at the University of Texas at Dallas (IACUC protocol #22–05), University of Texas Medical Branch Galveston (IACUC protocol # 2011111), and University of Guelph (CCAC AUP 4806, 4884) and were conducted in accordance with institutional and federal guidelines.

Female mice aged 7–10 weeks and weighing 16–23 g were used for all experiments. Wild-type C57BL/6J (WT), TdTomato reporter (B6.Cg-*Gt(ROSA)26Sor*^*tm9(CAG-tdTomato)Hze*^/J), and *Camp* KO (B6.129X1-*Camp*^*tm1Rlg*^/J) mice were obtained from The Jackson Laboratory and bred in-house under specific pathogen-free conditions. *Mrgprb2* KO, *MRGPRX2* transgenic, and *Mrgprb2*-cre mice were generated as previously described^23^. All mice were maintained under a 12 hours light/12 hours dark cycle with access to food and water at all times.

### Mouse urinary tract infection model

UTI model was performed on mice as previously described^4^. In short, the UPEC strain UTI89 was streaked onto LB agar plates. A single colony was inoculated into LB broth and cultured overnight at 37 °C under static aerobic conditions.

Bacterial cultures were adjusted to OD600 = 0.35 (~2 × 10^8^ CFU/mL) in sterile PBS. Mice were anesthetized with isoflurane and transurethrally catheterized to administer 50 μL of bacterial inoculum (1 × 10^7^ CFU) or sterile PBS (control).

Urine samples were collected at 1, 6, and 24 hpi. Mice were euthanized at 24 hpi using CO_2_ followed by cervical dislocation. Bladders and kidneys were aseptically dissected for further experiments.

### CFU enumeration

Bladders and kidneys were collected at 24 hpi and homogenized in 1 mL sterile PBS using 2 mL ceramic bead tubes (Fisherbrand, #15–340-154) and Bead Ruptor 4 homogenizer (OMNI International) at speed 5 for two cycles of 45 seconds with 1 min cooling on ice between cycles.

Homogenates and urine samples were serially diluted (10-fold) in sterile PBS and plated on LB agar plates. Colonies were enumerated after overnight incubation at 37 °C.

### Tissue Histology

Bladders were fixed in 4% paraformaldehyde at 4 °C for 48 hours, dehydrated sequentially in 20% and 30% sucrose in PBS, embedded in Optimal Cutting Temperature (OCT) compound, and frozen in liquid nitrogen. Cryosections of 10 μm were cut using a cryostat and mounted on charged slides. Sections were air-dried and then baked at 65 °C for one hour.

### Hematoxylin and Eosin staining

Bladder sections were washed in PBS to remove OCT compound and stained using the Vector Laboratories Hematoxylin and Eosin Stain Kit (H3502) according to the manufacturer’s instructions. Images were acquired using Keyence microscope. H&E images were quantified using ImageJ. Quantification was conducted by an experimenter blinded to experimental group.

### Avidin staining

Bladder sectioned were washed in PBS to remove OCT and incubated with Avidin-FITC (ThermoFisher Scientific; cat#A821) diluted 1:500 in PBS for 2 hours at room temperature. Avidin was washed off with PBS and coverslip was placed on slide before imaging it on confocal microscope.

### Flow cytometry

Bladders were minced and enzymatically dissociated in RPMI containing 2.5 mg/mL Liberase TL (Sigma-Aldrich; #5401020001) and 10% DNase I (Sigma-Aldrich; #DN25–1G) at 37 °C for 1 h with gentle rotation. Dissociated bladders were filtered through 70 μm cell strainers and washed with PBS.

Single-cell suspensions were stained with Zombie NIR Fixable viability dye (BioLegend; #423105) followed by surface staining with fluorophore-conjugated antibodies against CD45 (BioLegend; #103107), CD11b (BioLegend; #101255), and Ly6g (BioLegend; #127671). Samples were filtered through 40 μm strainers before data acquisition on a Sony FACS SH800Z flow cytometer. Data were analyzed using FlowJo software. Gating strategies are shown in Supplementary Fig. S3b.

### RNA extraction

Bladders were homogenized in 500 μL TRIzol reagent (Thermo Fisher Scientific; #15596026) using a handheld homogenizer. Lysates were centrifuged at 17,000 × g for 5 min at 4 °C. RNA was purified from 450 μL lysate using the Zymo Direct-zol RNA Miniprep Kit (Zymo Research; R2050) according to the manufacturer’s protocol. RNA concentration and purity were assessed by ThermoScientific Nanodrop One (cat# 13–400-525).

### Bulk RNA sequencing

RNA samples were submitted to SeqCenter (Pittsburgh, PA) for library preparation and sequencing. Poly(A)-enriched libraries were generated and sequenced on an Illumina NovaSeq × Plus platform to obtain 100 million paired-end reads per sample.

Raw Fastq reads were aligned to the *Mus musculus* reference genome (ENSEMBL GRCm39.113) using STAR^40^ (version 2.7.11b) with default parameters to generate BAM files. BAM files were analyzed using featureCounts^41^ (version 2.0.8) to generate gene count file with ENSEMBL IDs. Differential gene expression analysis was performed on counts file using DESeq2^42^ package in R. ENSEMBL IDs were aligned to gene names in R using org.Mm.eg.db and AnnotationDBI packages. Volcano plots were generated using EnhancedVolcano package in R. Genes lacking annotation were excluded from visualization but included in statistical analysis and PCA plot estimation. Gene Ontology enrichment analysis for biological pathways was performed using ClusterProfiler^25^ in R on genes with log2foldchange cutoff of 0.75 and p-value cutoff of 0.05.

### Single cell RNA sequencing analysis

Previously published ScRNA-seq on bladder immune cells (GSE25232)^21^ was obtained from GEO data base. Sample GSM7999426 was analyzed with R package Seurat^43^ with default settings.

### Quantitative PCR

cDNA was synthesized from RNA using the High-Capacity cDNA Reverse Transcription Kit (Applied Biosystems; #4368814). Quantitative PCR was performed using TaqMan assay on a QuantStudio 3 Real-Time PCR System (#A28567). Relative gene expression was calculated using the ΔCt method and normalized to housekeeping *Actb* gene.

### Osthole administration

Osthole was first dissolved in DMSO, then diluted in Tween 80, and then resuspended in PBS to achieve a final formulation of 10% Tween 80 and 10% DMSO in PBS. Vehicle control consisted of the same formulation of Tween 80 and DMSO but without osthole. Mice were anesthetized with isoflurane and administered 50 μL of osthole (10 mg/kg) or vehicle via transurethral catheterization.

### MIC assay on osthole

UTI89 was grown overnight at 37 °C in static conditions. This overnight culture was sub-cultured 1:100 into fresh LB and grown to mid-log phase (OD600 = ~0.4). The culture was then diluted in 10% Tween 80 in LB to achieve OD600 of about 0.002. 180μL of this diluted culture was added to wells containing 20μL osthole in DMSO in various concentrations that were achieved by two-fold dilution of the highest concentration in a 96-well plate. For comparison, no osthole was added to one of the well. Final formulation of all wells was osthole (except for no drug well) + 10% Tween 80 + 10% DMSO in LB. Plate was incubated at 37 °C in static conditions for 20 hours and OD600 was measured using microplate reader with LB as blank. MIC was determined by comparing the bacterial growth in no drug well to wells containing the drug.

### Mast cell degranulation assay

Mouse PCMC were obtained from WT and *Mrgprb2* KO mice as previously described^44^. WT and *MRGPRX2* KO LAD2 mast cells were cultured and maintained as previously described^16^. Mast cell degranulation was measured using beta-hexosaminidase assay with LL-37 and CRAMP on LAD2 cells and PCMC as previously described^16^.

### Data analysis and statistics

All statistical analyses were performed using GraphPad Prism. Normality was assessed using the Shapiro-Wilk test. For normally distributed data, Two tailed student’s t-test or one-way ANOVA test were used. For not normally distributed data, Two-tailed Mann-Whitney U test or Kruskal-Wallis test were performed. Unless otherwise stated, data are presented as median ± 95% CI. Statistical significance was defined as p < 0.05. Exact p values are reported in the figures. All experiments in this study were repeated independently at least three times with exception of RNAseq.

## Supplementary Material

All supplementary information is attached to the manuscript

This is a list of supplementary files associated with this preprint. Click to download.

• Supplementmergedupdated.pdf

## Data Availability

RNAseq data generated in this study has been deposited at Gene Expression Omnibus (GEO) and is publicly available under the accession number GSE324031. The previously published single-cell-RNAseq data set reanalyzed in this study is publicly available at GEO under the accession number GSE252321^21^.
